# Supplementary material for: Effects on health-related quality of life in the randomized, controlled crossover trial ADIRA (Anti-inflammatory Diet In Rheumatoid Arthritis)
Source: PLoS One. 2021 Oct 14;16(10):e0258716. doi: 10.1371/journal.pone.0258716 (PMC8516209; doi:10.1371/journal.pone.0258716)
Supplement: S1 Table — Modelled estimates of differences in effects of a proposed anti-inflammatory diet (intervention) compared to a diet nutritionally alike usual Swedish intake (control) in patients with rheumatoid arthritis in the randomized controlled crossover trial ADIRA, with imputed values for missing data due to drop-out. (PDF) [file pone.0258716.s001.pdf]

S1 Table. The effects on HrQoL in the ADIRA trial (sensitivity analyses). Modelled estimates of differences in effects of a proposed anti-inflammatory diet (intervention) compared to a diet nutritionally alike usual Swedish intake (control) in patients with rheumatoid arthritis in the randomized controlled crossover trial ADIRA, with imputed values for missing data due to drop-out[a]

	**Worst-case scenario[b]**		**Median[c]**		**Best-case scenario[b]**	
	**Mean difference (95% CI)**	**p-value**	**Mean difference (95% CI)**	**p-value**	**Mean difference (95% CI)**	**p-value**
**HAQ[d]**	-0.035 (-0.152, 0.082)	0.546	-0.040 (-0.156, 0.076)	0.491	-0.050 (-0.166, 0.066)	0.393
**SF-36[d]**						
Physical Functioning	5.066 (-0.561, 10.692)	0.077	5.280 (-0.324. 10.884)	0.064	5.709 (0.104, 11.315)	0.046
Role-Physical	0.065 (-7.718, 7.849)	0.987	0.322 (-7.443, 8.087)	0.934	0.579 (-7.185, 8.342)	0.881
Bodily Pain	0.941 (-4.811, 6.694)	0.746	1.199 (-4.523, 6.921)	0.678	1.649 (-4.072, 7.371)	0.568
General Health	-3.603 (-8.508, 1.302)	0.145	-2.938 (-7.790, 1.914)	0.228	-2.625 (-7.507, 2.258)	0.284
Physical Component Summary	-0.043 (-2.255, 2.169)	0.969	0.096 (-2.103, 2.296)	0.931	0.299 (-1.912, 2.510)	0.789
Vitality	-3.362 (-10.422, 3.698)	0.343	-3.098 (-10.167, 3.971)	0.382	-2.698 (-9.817, 4.421)	0.449
Social Functioning	-0.719 (-8.155, 6.717)	0.848	-0.719 (-8.155, 6.717)	0.848	-0.181 (-7.625, 7.263)	0.962
Role-Emotional	3.445 (-2.346, 9.235)	0.237	3.445 (-2.346, 9.235)	0.237	4.022 (-1.664, 9.708)	0.161
Mental Health	1.518 (-3.994, 7.030)	0.581	1.927 (-3.503, 7.357)	0.478	2.128 (-3.285, 7.542)	0.432
Mental Component Summary	0.160 (-2.751, 3.070)	0.912	0.317 (-2.570, 3.204)	0.826	0.567 (-2.318, 3.452)	0.694
**VAS Pain (mm)[d]**	-1.740 (-10.986, 7.506)	0.706	-2.424 (-11.667, 6.819)	0.600	-3.094 (-12.429, 6.242)	0.508
**VAS Fatigue (mm)[d]**	-1.397 (-10.772, 7.978)	0.765	-1.893 (-11.149, 7.363)	0.682	-2.788 (-11.950, 6.375)	0.543
**VAS Morning stiffness (mm)[d]**	2.356 (-5.169, 9.882)	0.536	1.745 (-5.721, 9.211)	0.644	1.483 (-5.985, 8.951)	0.694
**Morning stiffness (min)[d]**	4.242 (-9.044, 17.528)	0.528	3.599 (-9.666, 16.864)	0.591	3.171 (-10.113, 16.454)	0.637

ADIRA, Anti-inflammatory Diet In Rheumatoid Arthritis; HAQ, Health Assessment Questionnaire; HrQoL, Health-related Quality of Life; SF-36, 36-item Short Form Health Survey; VAS, Visual Analogue Scale

[a] Differences at the end of diet periods (Intervention – Control), n = 50

[b] Missing post-values replaced with median difference of control periods added to the participants' pre-value if control period, or lower/higher quartile difference of intervention periods if intervention period.

[c] Missing post-values replaced with period-specific median difference added to the participants' pre-value.

[d] Linear mixed model with period, treatment, sequence and baseline value as fixed effects and subject as random effect
