# Supplementary material for: Effects on health-related quality of life in the randomized, controlled crossover trial ADIRA (Anti-inflammatory Diet In Rheumatoid Arthritis)
Source: PLoS One. 2021 Oct 14;16(10):e0258716. doi: 10.1371/journal.pone.0258716 (PMC8516209; doi:10.1371/journal.pone.0258716)
Supplement: S2 Table — Modelled estimates of differences in effects of a proposed anti-inflammatory diet (intervention) compared to a diet nutritionally alike usual Swedish intake (control) in sensitivity analyses in the randomized controlled crossover trial ADIRA. (PDF) [file pone.0258716.s002.pdf]

S2 Table. The effects on HrQoL in the ADIRA trial (sensitivity analyses). Modelled estimates of differences in effects of a proposed anti-inflammatory diet (intervention) compared to a diet nutritionally alike usual Swedish intake (control) in sensitivity analyses in the randomized controlled crossover trial ADIRA[a].

	**Completers[b]**		**Compliers[c]**		**Generalized logistic mixed model[d]**						
					**Intervention**		**Control**		**Intervention - Control**		
	**Mean diff. (95% CI)**	**p-value**	**Mean diff. (95% CI)**	**p-value**	**<Median, %**	**SE**	**<Median, %**	**SE**	**Diff.**	**95% CIs**	**p-value**
**HAQ[e]**	-0.049 (-0.174, 0.076)	0.436	-0.002 (-0.130, 0.126)	0.978	39.7	10.7	38.2	10.2	1.5	-26.1, 29.0	0.915
**SF-36[e]**											
PF	5.507 (-0.564, 11.577)	0.075	5.697 (-0.506, 11.900)	0.071	35.4	11.3	58.2	10.9	-22.8	-54.7, 9.1	0.160
RP	0.182 (-8.272, 8.635)	0.996	-0.677 (-9.735, 8.380)	0.880	46.2	10.2	51.6	9.6	-5.3	-31.3, 20.7	0.685
BP	1.285 (-4.869, 7.439)	0.679	0.724 (-5.697, 7.144)	0.823	47.9	9.5	45.8	9.6	2.1	-24.7, 28.9	0.874
GH	-3.765 (-8.806, 1.276)	0.138	-3.944 (-8.675, 0.788)	0.100	55.3	10.7	42.3	10.2	13.0	-16.7, 42.7	0.388
PCS	-0.114 (-2.503, 2.275)	0.925	0.046 (-2.404, 2.496)	0.970	44.7	11.0	53.1	10.7	-8.4	-38.9, 22.2	0.586
VT	-3.414 (-11.025, 4.198)	0.370	-3.119 (-11.044, 4.806)	0.436	49.0	10.3	44.8	9.0	4.3	-23.0, 31.5	0.755
SF	-1.331 (-9.403, 6.740)	0.744	-1.656 (-9.881, 6.570)	0.690	48.5	9.5	51.3	8.7	-2.8	-27.9, 22.4	0.828
RE	4.503 (-1.550, 10.556)	0.140	2.249 (-4.646, 9.144)	0.513	38.0	10.2	47.0	10.3	-9.1	-34.5, 16.4	0.481
MH	1.683 (-3.893, 7.259)	0.544	0.604 (-5.080, 6.287)	0.831	51.8	11.6	52.9	11.4	-1.1	-30.1, 28.0	0.942
MCS	0.432 (-2.602, 3.466)	0.775	-0.083 (-3.260, 3.095)	0.958	62.0	11.8	52.5	11.3	9.5	-18.7, 37.6	0.504

**VAS Pain (mm)[e]**	-1.629 (-11.593, 8.335)	0.742	-1.952 (-12.318, 8.413)	0.705	51.8	8.4	46.3	8.1	5.5	-17.4, 28.4	0.633
**VAS Fatigue (mm)[e]**	-2.230 (-12.005, 7.545)	0.646	-0.064 (-10.000, 9.872)	0.990	47.1	8.3	50.7	8.0	-3.6	-26.6, 19.4	0.757
**VAS Morning stiffness (mm)[e]**	2.620 (-5.436, 10.676)	0.520	4.638 (-3.421, 12.697)	0.255	46.6	9.0	53.5	9.1	-6.9	-32.5, 18.7	0.592
**Morning stiffness (min)[e]**	4.754 (-9.614, 19.123)	0.512	7.922 (-6.388, 22.233)	0.274	44.9	8.8	43.3	8.7	1.6	-22.6, 25.9	0.894

ADIRA, Anti-inflammatory Diet In Rheumatoid Arthritis; Diff., Difference; HAQ, Health Assessment Questionnaire; HrQoL, Health-related Quality of Life; SE, Standard error; SF-36, 36-item Short Form Health Survey (*PF, Physical Functioning; RP, Role-Physical; BP, Bodily Pain; GH, General Health; PCS, Physical Component Summary; VT, Vitality; SF, Social Functioning; RE, Role-Emotional; MH, Mental Health; MCS, Mental Component Summary)*; VAS, Visual Analogue Scale.

[a] Values represent the end of diet periods. Differences = Intervention – Control
[b] Including only participants completing both diet periods (n = 44)
[c] Including only diet periods deemed to have been performed with good compliance to the diets (n = 47)
[d] Including participants completing ≥1 diet period (n = 47).
[e] Linear mixed model with period, treatment, sequence and baseline value as fixed effects and subject as random effect
