# Supplementary material for: Effects on health-related quality of life in the randomized, controlled crossover trial ADIRA (Anti-inflammatory Diet In Rheumatoid Arthritis)
Source: PLoS One. 2021 Oct 14;16(10):e0258716. doi: 10.1371/journal.pone.0258716 (PMC8516209; doi:10.1371/journal.pone.0258716)
Supplement: S3 Table — Modelled estimates of differences in effects of a proposed anti-inflammatory diet (intervention) compared to a diet nutritionally alike usual Swedish intake (control) in patients with rheumatoid arthritis in the randomized controlled crossover trial ADIRA. (PDF) [file pone.0258716.s003.pdf]

S3 Table. The effects on HrQoL in the ADIRA trial. Modelled estimates of differences in effects of a proposed anti-inflammatory diet (intervention) compared to a diet nutritionally alike usual Swedish intake (control) in patients with rheumatoid arthritis in the randomized controlled crossover trial ADIRA[a]

	Mean difference between diet periods[b]	Standard Error	Degrees of freedom	t-value	p-value	95% CIs	AIC
**HAQ[c]**	-0.041	0.060	39.88	0.675	0.503	-0.162, 0.081	69.760
**SF-36[c]**							
Physical Functioning	5.392	2.974	86.00	-1.813	0.073	-0.520, 11.304	728.626
Role-Physical	0.602	4.054	40.80	-0.148	0.883	-7.586, 8.790	789.239
Bodily Pain	1.499	3.004	86.00	-0.499	0.619	-4.472, 7.471	729.710
General Health	-3.161	2.484	34.40	1.272	0.212	-8.208, 1.885	704.312
Physical Component Summary	0.019	1.166	86.00	-0.016	0.987	-2.300, 2.338	565.269
Vitality	-2.973	3.679	40.07	0.808	0.424	-10.408, 4,463	767.737
Social Functioning	-0.582	3.946	86.00	0.148	0.883	-8.427, 7.263	777.098
Role-Emotional	4.057	2.955	39.25	-1.373	0.178	-1.920, 10.033	764.229
Mental Health	1.406	2.713	36.49	-0.518	0.608	-4.094, 6.906	721.110
Mental Component Summary	0.344	1.471	39.55	-0.234	0.816	-2.630, 3.318	618.014
**VAS Pain (mm)[c]**	-2.466	4.793	40.95	0.515	0.610	-12.145, 7.213	812.785
**VAS Fatigue (mm)[c]**	-2.554	4.709	39.86	0.542	0.591	-12.073, 6.966	811.620
**VAS Morning stiffness (mm)[c]**	1.718	3.970	86.00	-0.433	0.666	-6.174, 9.610	777.896
**Morning stiffness (min)[c]**	3.748	7.033	86.00	-0.533	0.595	-10.233, 17.730	877.293

ADIRA, Anti-inflammatory Diet In Rheumatoid Arthritis; AIC, Akaike's Information Criterion; HAQ, Health Assessment Questionnaire; HrQoL, Health-related Quality of Life; SF-36, 36-item Short Form Health Survey; VAS, Visual Analogue Scale

[a] n = 47

[b] Differences at the end of diet periods (Intervention – Control)

[c] Linear mixed model with period, treatment, sequence and baseline value as fixed effects and subject as random effect
