# Supplementary material for: Effects on health-related quality of life in the randomized, controlled crossover trial ADIRA (Anti-inflammatory Diet In Rheumatoid Arthritis)
Source: PLoS One. 2021 Oct 14;16(10):e0258716. doi: 10.1371/journal.pone.0258716 (PMC8516209; doi:10.1371/journal.pone.0258716)
Supplement: S2 File — (PDF) [file pone.0258716.s005.pdf]

 Study protocol approved by the ethical committee

ADIRA (Anti-inflammatorisk kost vid reumatoid artrit): en randomiserad cross-over studie av effekten på sjukdomsaktivitet och livskvalitet

ADIRA (Anti-inflammatory diet in rheumatoid arthritis): a randomized cross-over trial of the effect on disease activity and quality of life

Abstract

Rheumatoid arthritis (RA) is a chronic inflammatory disease that affects 0.5-1% of the population, and where many patients in spite of modern pharmacological treatment fail to reach remission. This affects physical as well as mental wellbeing and leads to severely reduced quality of life and reduced work capacity, thus yielding high individual as well as societal costs. To optimize treatment, alternatives such as diet should be evaluated as complement to pharmacological treatment. The main goal of the randomized cross-over trial ADIRA (Anti-inflammatory Diet In Rheumatoid Arthritis) is to test the hypothesis that a diet intervention will decrease disease activity and costs and improve quality of life in patients with established RA. In total, 80 RA patients with moderate disease activity will be randomized to receive initially either a portfolio diet based on several food items with suggested anti-inflammatory effects or a control diet (western type), during 2 x 10 wks with a 3 months wash-out between diets. Both groups continue with regular pharmacological treatment. Known food biomarkers will be analyzed to measure intervention compliance. Impact on disease activity (measured by DAS28, a composite score which predicts disability and progression of RA) and quality of life is evaluated after each diet regimen. Metabolomics will be used to evaluate the potential to predict responders to dietary treatment. ADIRA will provide evidence whether dietary treatment of RA leads to more patients reaching remission and improved quality of life and work capacity as well as reduce individual and societal costs. Scientific evidence exists for anti-inflammatory effect by single foods on RA, but no study exists where these foods have been combined to obtain maximum effect and thus have the potential to offer a substantial improvement in patient life quality. Such evidence has been asked for by RA patents as well as by treating physicians.

Project description

Main goal and specific aims

The main goal of the proposed project is to develop an anti-inflammatory diet to optimize treatment for patients with rheumatoid arthritis (RA). Specific aims are to investigate the effect of a portfolio diet intervention on inflammation and disease symptoms, quality of life and costs to the individual as well as society and to use metabolomics to predict responders to the intervention.

A large proportion of patients with RA have remaining high disease activity, despite modern pharmacological treatment. Therefore, complementary treatment options including dietary treatment are called for by patients and treating physicians. Today, due to lack of evidence for disease specific recommendations, patients with RA are referred to the same dietary guidelines as the general population. However, studies indicate that many patients with RA exclude food or food groups by their own initiative and also that the dietary intake in general is of poor quality. Hence, there is an urgent need to establish if diet can benefit patients with RA, and if so which diet is most beneficial. Poor dietary quality in itself is a risk factor for many non-communicable diseases, and these are over-represented among patients with RA. Thus, improvement in dietary quality could not only reduce the disease activity of RA but also improve long-term health of this vulnerable group.

Our project in relation to the state of the art in the area

Rheumatoid arthritis is a chronic autoimmune disease, characterized by systemic inflammation and joint damage. It affects 0.5-1% of the population in Sweden and globally. About two-thirds of those afflicted are women and women also experience higher disease severity, with more pain and tender joints. Early diagnosis and treatment is pivotal as the disease progresses with joint erosion and function loss as consequences of the inflammatory process. Many patients have persistent inflammation that contributes to joint destruction in spite of ongoing pharmacological treatments[1]. The inflammatory process also causes fatigue and inflammatory-reduced quality of life. The inflammation leads to increased risk for cardiovascular events and together with the immune-suppressive treatment also to increased risk for infections--the two leading causes of death among patients with RA[2]. Patients with RA face approximately 50% increased risk of myocardial infarction and stroke compared to the general population and their risk of CVD is comparable to that of patients with diabetes[3,4]. Consequently, life-expectancy among patients with RA is reduced with 5-10 years, compared to non-diseased individuals.

The long-term consequences of RA depend on the time of initiation and effectiveness of treatment, in terms of limiting the inflammatory process. Any remaining inflammation likely contributes to the development of atherosclerosis and premature death among RA patients[5]. Also, traditional risk factors of CVD like dyslipidemia, hypertension, smoking and physical inactivity are likely to contribute. Chronic inflammation may further lead to altered body composition with lower levels of lean mass (mainly muscle) combined with increased levels of fat mass. This condition is referred to as rheumatoid cachexia, and can be difficult to detect as body mass index and weight often still are within the normal ranges. A Swedish study found that more than 50% of RA patients were overweight (BMI ≥25 kg/m^2) and 20% were cachectic[6]. The reduction in muscle mass may add to the physical deterioration that accompanies RA, while the increased fat mass could add to the increased risk of CVD.

The treatment of patients with RA aims at long-term remission i.e. no pain, tenderness of joints or functional impairment. This is achieved in 10-50% of patients with early RA[7]. Today’s therapy includes immunosuppression by disease modifying anti rheumatic drugs, steroids and the more recent line of biological therapies such as TNF-α-inhibitors[8]. However, a recent systematic review highlighted that, in spite of existing therapies, a substantial proportion of

patients with RA still experience severely impaired health[9]. The inflammation leads to joint destruction and fatigue as well as to impaired physical functioning, work productivity and activities of daily living, and compromise overall emotional well-being. A negative impact on relationships with friends and family has been reported by one-fifth of patients with RA. Further, suboptimal mental health is reported in a substantial proportion of RA patients[9].

RA thus continues to present a considerable human and economic burden. An estimated one-third of patients with RA terminate employment prematurely, and 5 years after diagnosis 30-40% of patients experience work disability[9]. During the year 2010, the total annual economic burden of RA in Sweden was estimated to SEK 6 billion; almost 60% of this cost was related to the indirect cost of sick leave and early retirement[10]. Likewise, the total annual economic burden of RA has been estimated to EUR 45.3 billion in Europe and EUR 41.6 billion in the US[9]. Direct costs associated with RA include medications, hospitalizations, clinic visits, laboratory monitoring imaging, toxicity and medical assist devices. Indirect costs include loss of earnings and caregiver productivity as well as costs due to pain, depression and anxiety.

The authors of the systematic review conclude that "Despite advances in treatment that have helped to improve outcomes for patients with RA, treatment goals, aspirations, and expectations are seldom met for both patients and physicians. Novel treatment approaches for RA need to be tested for their ability to ameliorate contemporary unmet need."[9]

Also in Sweden, major suffering among patients with RA exists. As many as 45% still have moderate to high disease activity, reflecting serious weaknesses in their treatment and care (Swedish Rheumatology Quality Register http://srq.nu/). This situation may be because of insufficient pharmacological treatments, poor compliance, poor response to prescribed treatment and/or lack of other additional treatment modalities such as lifestyle changes. Thus, there is an urgent need for improved treatment of patients with RA, including diet and lifestyle changes.

In 2015, the Global Burden of Disease Project again demonstrated that dietary risks (including low fruit, low whole grains, high dietary salt) contributed to the largest proportion of disability-adjusted life-years (DALYs) globally for both sexes, thus being more important than high blood pressure, high body mass index or smoking[11]. Further, in high income countries, WHO estimates that high blood pressure, high blood glucose, high cholesterol, physical inactivity, overweight and obesity and low intake of fruit and vegetables cause 25% of all deaths[12]. Associations between diet and chronic diseases such as cardiovascular diseases, cancer and diabetes have been established. Hence, for these diseases evidence based dietary treatment guidelines are available. In contrast, for inflammatory diseases such as RA no dietary guidelines exist, reflecting the ambiguous evidence base. Further research is thus needed, as patients with RA often ask their treating physicians for diet advice as a complement to pharmacological treatment[13].

The cause of RA is unknown, but both genetic, environmental and lifestyle factors are likely to contribute[14]. Smoking is a risk factor[15] and moderate alcohol intake and physical activity are associated with decreased risk[16]. The potential for diet to improve RA is obvious; diet could influence symptoms of RA by

influencing the inflammatory activity, changing the lipid profile, increasing antioxidant levels, and altering the microflora of the intestine. Many nutrients and dietary components are related to the human immune system or to inflammation[17]. Some are co-factors in immune- or inflammatory response, such as zinc. Others are antioxidants, eg selenium, vitamins E and C.

RA has been associated with low serum concentrations of zinc, selenium, vitamins D and B6 although some of this may reflect inflammatory response[18]. Long chain fatty acids from fish have shown to reduce morning stiffness, number of tender or swollen joints and pain [19]. Studies on the effect of probiotics for prevention or treatment of RA indicate improved function and decreased disease activity[20].

Due to lack of evidence, there are no dietary guidelines or recommendations for patients with RA, and they are encouraged to follow the same recommendations as the general population. These general recommendations aim at increasing intake of fruit and vegetables (to 500 g/day), fish and shellfish (to 2-3 times per week), choosing whole grain over refined grain products and reduce fat dairy products over full-fat varieties. Also, intakes of red meat and refined sugars should be reduced[21].

For decades patients have used different diets to try to improve the symptoms of RA and dietary manipulation is still widely used today. A study among Finnish women with RA showed that 40% believed diet contributed to their disease and 51% had changed their dietary habits since diagnosed[22]. The most common change was to reduce the intakes of red meat and fat, and the reason was the hope of a cure. An American study showed that women with RA scored lower on the Healthy Eating Index than women without RA, indicating an overall poorer dietary quality among RA patients[23]. Women with RA had a lower intake of fruit, whole grain, fiber and oil. Also, as RA often affects the smaller joints in the hands, the ability to prepare food may be impaired.

Few studies have investigated the effects of dietary treatment on inflammation and symptoms among patients with RA. Observational studies show lower incidences of RA among people with larger consumption of n-3 fatty acids[24] and fish[25]. Intervention studies using complete diets show that the Mediterranean diet (characterized by high intakes of olive oil and fish and low intake of red meat and dairy products) have some positive effects at alleviating pain among patients with RA[26,27]. Also, a low-fat vegan diet has been beneficial[28] as well as a diet rich in unsaturated fat with little saturated fat[29].

Interventions with individual food components show that marine n-3 fatty acids have positive effects with the potential to modify the immunological reactions in RA[30]. Also, a double-blind placebo-controlled study found that probiotics improved the profile of cytokines IL-10, IL-12 and TNF-α in comparison with placebo[31].

Dietary treatment in RA may also have beneficial effects on risk factors for CVD. Studies indicate that increased vegetable intake may improve the arterial function in patients with RA, thus possibly improving long-term health and survival. A similar effect of an increased intake of marine n-3 fatty acids is also believed to have a positive effect on CVD risk[32].

In conclusion, studies indicate that several diets and dietary components may have positive effects on clinical outcomes of RA, including perceived pain and inflammation. So far, no study has combined all the components with indicative effects on RA, thus limiting the possibility to evaluate the full potential of dietary treatment of the disease. Not only does dietary treatment have the possibility to decrease disease activity and inflammation, but it can also reduce risk factors for future cardiovascular events, thus promoting health in both the short and long-term. This could have impact, not only on the quality of life and health of the individual patients, but also reduce the costs of medical care and patients' ability to remain employed. In sum, high-quality studies that evaluate the combined effect of foods rich in anti-inflammatory and immune strengthening substances that could interact synergistically on RA are needed.

Improved treatment of RA likely reduces costs to the individual and to society in that health-related quality of life likely improves, sick leave and early retirement may be reduced and in the long run expected life years may increase. Changes in diet may of course affect individual costs for purchasing and preparing their food. Still, diet intervention in RA constitutes a novel treatment approach that may have high cost benefit due to its low adverse event rate and low cost, in comparison with new and potent pharmacological treatments.

Whether RA patients respond to a dietary intervention or not might relate to genetics, habitual diet and/or the diversity and intensity of disease activity. Metabolomics, ie the analysis of low molecular weight metabolites, investigates overall metabolomic activity, taking genetic and environmental variation into account. It is a powerful tool to measure global and dynamic metabolic responses in disease and clinical intervention[33] and some research also exist on RA. Different baseline metabolic profiles have been identified among patients with active RA compared to those in remission[34]. Further, metabolomic analysis in RA patients attempt to relate metabolites to different symptoms and to identify subgroups that might respond or not to a specific treatment. For example, metabolites that predict response to methotrexate treatment have been identified[35]. Thus, it may be possible to identify metabolic biomarkers to predict response also to dietary treatment among RA patients.

Diet contains many known and unknown potent substances. Thus, characterizing the entire metabolic finger print after a diet intervention could help in understanding what component in the diet that causes an effect, and also indicate which subjects who respond to such dietary treatment. The metabolomics methods are relatively new and therefore few dietary interventions have used this approach to look at metabolic effects.

Further, since the metabolism often is changed in RA patients due to the metabolic syndrome or cachexia, metabolomics opens new possibilities to look at previously unknown metabolic changes as a consequence of a specific diet. Our proposed project will be able to evaluate how diet influences markers such as acylcarnitine and pantothenic acid; markers that both are provided by diet and produced endogenously and that are thought to relate to muscle breakdown and fatigue syndrome[36].

Study design and methods

A randomized controlled cross-over trial (ADIRA, Anti-inflammatory Diet In Rheumatoid Arthritis) will be carried out among established RA patients with moderate disease activity to evaluate response to a portfolio diet treatment, compared to control diet (i.e. western diet). Both groups will continue with pharmacological treatment as usual. In Sweden, patients with established RA are treated with disease modifying anti rheumatic drugs and biologics at out-patient clinics according to the Swedish Society for Rheumatology Guidelines[37]. The portfolio diet treatment builds on a combination of individual food items with indicative effects on different RA symptoms. The trial thus evaluates the treatment potential of a diet based on a combination of functional concepts that likely potentiate each other in their anti-inflammatory effects. This concept has been successful in improving cardiometabolic risk parameters among healthy overweight adults[38]. Although it is not possible to fully blind a diet intervention, the intervention and control groups will be treated as similarly as possible except for diet content. Assessors of outcome will be blinded.

RA patients residing in the Västra Götaland region will be identified through the Swedish Rheumatology Quality Register (http://srq.nu/). Hence, unique information about disease status in relation to treatment is here available. In total, 40 men and 40 women with established RA, >18 years of age, disease duration >2 years, moderate disease that is clinically stable and under adequate control and medication, will be invited to participate. Moderate disease activity translates to DAS28-SR >2.6 at screening and inclusion and pain in ≥ 3 joints. DAS28 stands for Disease Activity Score, where swelling and tenderness in 28 different joints are evaluated in combination with information on ESR or C-reactive protein (hs-CRP) and patient-reported global assessment of health. Exclusion criteria include other condition demanding active medical attention, changes in rheumatic medication last 3 months, intolerant or allergic to food items included in the intervention diets or not willing to eat omnivore diet.

Participants will be recruited Jan 2017– Dec 2018. Letters will be sent to 400 patients; a 20% response rate is expected based on previous experience. Responders will be invited to a screening visit where DAS28 is measured and study details presented. Patients fulfilling all inclusion criteria are thereafter invited to baseline measurements (see Fig 1). These include clinical phenotype and health assessment, 3 day food record, serum samples for metabolomics and inflammation markers (hs-CRP, ESR), Health Assessment Questionnaire (HAQ), Quality of Life (Qol) and a questionnaire on socio-demographics, lifestyle, acute health care visits and NSAID and corticosteroid consumption.

Thereafter, patients are randomized to either diet regimen for 10 weeks. After a 3-mo wash-out period, the alternative diet regimen is followed for another 10 weeks. For both diets, food bags will be delivered weekly by a home food delivery chain (Mat.se) and make up 50% of the daily intake. Participants in both groups will be provided with 5 main meals, 5 breakfast meals and 5 in-between-meal snacks per week. Main meals comprise of one ready-to-eat meal, and ingredients for four easily prepared meals to cook. All participants are encouraged to say weight stable and will receive instructions on how to achieve this. Participants are also provided with recipes and information on the diet they are to follow. A brochure will be provided with inspiration for suitable other meals and foods to supplement to the provided meals during each intervention.

The anti-inflammatory diet bag will contain 5 main meals per week: fish 2-3 times weekly, mainly oily fish, and 2-3 vegetarian meals rich in prebiotics. Breakfast meals consist of low-fat dairy products, wholegrains, vegetables and berries and probiotic fruit juice is included with each breakfast meal. In-between-meal snacks comprise of 2 fruits per day. Participants are also provided with olive/rapeseed oil for cooking, and margarine as spread (see Fig 2). Wholegrain, legumes and vegetables ensure an adequate intake of fiber and prebiotics, and fish, oils and nuts provide unsaturated and omega 3 fatty acids and probiotics are added to the diet. Participants are not provided with any meat and are encouraged to restrict intake of red meat to 3 servings per week. This diet therefore combines components of a vegetarian and Mediterranean diet with omega 3 fatty acids and probiotics, which all have shown promising effects on clinical outcomes of RA.

The control diets main meals consist of one ready-to-eat meal and 4 easily prepared meals with meat or chicken. The 5 servings of breakfast meals consist of full-fat dairy products, corn flakes, white bread and fruit juice without probiotics. In-between-meal snacks comprise of yoghurt/curd and crisp bread sandwiches. Participants will also be provided with butter for cooking and butter for spread, together with recipes and instructions for food preparation. The control diet mimics the nutritional intake from the national dietary survey Riksmaten 2010. Here, the control diet reflects the reported intakes of men and women between the ages of 45-65 in terms of macronutrients and quality of fat and carbohydrate intake. Therefore, the diet will be representative of the dietary habits of the average Swedish person.

Compliance to the diets will be monitored using objective biomarkers week 10 and 24-h diet recalls at week 5 in both periods.

Analysis of outcome
Outcomes will be measured after each diet regimen. Primary outcome is DAS28, where we expect a difference between diet regimens >0.6 units (considered clinically relevant). Secondary outcomes include HAQ, Qol, inflammation markers, acute health care visits and NSAID and corticosteroid consumption, body composition (Bioelectric Impedance Analysis) and pain. Changes in outcomes will be compared for the two diet regimens in multivariable regression analyses, adjusting for baseline disease activity values and confounders (eg age, other lifestyle factors). Interaction by sex and socio-economic position also will be evaluated. Cost-effectiveness of anti-inflammatory diet compared to control diet will be evaluated according to standard procedures[39-41].

Sample size is based on Skoldstam[27], where 30 patients per group in a parallel trial of Mediterranean diet were sufficient to detect a difference of 0.6 units DAS28. In ADIRA, we account for a patient group with somewhat lower disease activity, improved pharmacological treatments of today and a drop-out and non-adherence rate of 30%, based on our previous experience. ADIRA is a cross-over trial with each patient being its own control and the intervention is based on foods rich in anti-inflammatory and immune strengthening substances that may act synergistically. These features likely increase the ability to identify a significant difference.

For metabolomics, serum samples will be analyzed with nuclear magnetic resonance (NMR) at the Swedish NMR Centre at University of Gothenburg as well as with mass spectrometry (MS) at Chalmers Technical Institute, Gothenburg. We will use an untargeted metabolomics approach for a global metabolite description using unsupervised statistical analyses, ie principal component analysis (PCA). Subsequently supervised clustering, ie orthogonal projection to latent structures-discriminant analyses (OPLS-DA) will be performed, to compare responders and non-responders. Finally, in a targeted metabolomics approach, metabolites providing the greatest discrimination between groups of responders vs. non-responders will be identified using publicly and commercially available as well as in-house developed databases. Our research group has worked with these methods for several years.

Work plan

During late autumn 2016, patients who meet the inclusion criteria are identified through the Swedish Rheumatology Quality Register (http://srq.nu/) and are asked by letter to participate in the study. We estimate it to be about 700 patients that will be invited by letter to the study and 80 persons are expected to participate. This allows for a dropout rate of 25% as 60 are needed for statistical power. The study will be carried out during two time periods with 50% of the participants in each time-period, for logistical reasons and to avoid contamination of the study protocol by season or holidays.

The first period group (N=40) will be recruited and screened during winter 2017 (see Fig 1). Subjects that show an interest in participating and fulfill the pre-inclusion criteria (see questions in "intresseanmälan" bilaga 5c) will be asked to leave a blood sample for SR and CRP, which is routine for this patient group, at Clinical Chemistry, Sahlgrenska University and will be booked for the inclusion visit. At screening we will make sure that the patients have read the written information and give them oral information before they sign a written consent. They will fill in a lifestyle questionnaire, get information about a three day food registration, be weighted, measured and their joints will be examined by a trained nurse. Participants that are included will be randomized to one of the two diets and start the intervention during weeks 7-8. A ten week dietary intervention will follow and thereafter a three month wash-out period during the summer. After the summer they will switch to the other diet for 10 weeks. During fall 2017, the second period group (N=40) is recruited and screened. The participants are randomized and start the first diet in week 34. Wash-out follows for 3 months, and the second dietary regime starts during week 5, 2018. Before and after each diet, baseline and endpoint measures will be taken.

All measurements and tests are performed at The Department Rheumatology and Inflammatory Research by registered nurses experienced in rheumatology assessments. All meals and ingredients included in the interventions are provided to the participants and delivered to their homes by a home delivery chain (mat.se). Recipes and information about the diet is also provided. The dietary protocol is developed by a team of nutrition professionals (nutritionist, dietitian) at the Department of Internal Medicine and Clinical Nutrition to ensure dietary quality, feasibility and compliance. All meals are easily prepared with minimal peeling and cutting that could be challenging during active phases of RA. The meals are also varied in style and ingredients to promote compliance and to

ensure satisfactory nutrient intake. Compliance to the diets is measured by interviews regarding the dietary intake of the last 24 hours (24-hour recalls) at weeks 5 and 10 of the interventions, and by analyzing biomarkers of intake.

After the study is completed, metabolomic analyses will be performed to identify biomarkers that might predict the response to a dietary intervention, and predict who would benefit from such a treatment. As the metabolites identified by metabolomic analyses are downstream of genetic, environmental and lifestyle factors, it provides the best assessment of an individual's likelihood of success of such an intervention.

Collaborative structure
The Dept Internal Medicine and Clinical Nutrition has a long history of assessing nutrition intake, running dietary trials and sampling biofluids. Winkvist, professor in nutrition, has extensive experience of running RCTs with diet interventions and is PI of a program in Nutritional Metabolomics. Ellegård, associate professor in clinical nutrition and senior physician, has extensive experience of research on the effects of diets on human metabolism. Lindqvist is assistant professor in food science and experienced in RCTs on diet interventions and food science.

The Dept Rheumatology and Inflammatory Research has a long tradition of rheumatology treatment and research. Gjertsson, professor in rheumatology, represents expertise in diagnostics, treatment and care of RA patients.

Hagberg, PhD in public health sciences and health economist at Region Örebro County, Univ Health Care Research Center and affiliated with Örebro Univ, has experiences of health economic evaluations in general and of economic evaluations of lifestyle interventions in specific. Hagberg and Winkvist have previously collaborated on health economic evaluation of diet interventions.

The Swedish NMR Centre is world leading in its equipment and a national research infrastructure for metabolomics. Dept Food Science, Chalmers Technical Institute, is one of three main centres for food and nutrition research in Sweden. It recently launched the Chalmers Metabolomics Centre, where MS metabolomics analyses are performed. Chemometric analyses will be carried out jointly with BILS (Bioinformatics Infrastructure for Life Sciences), a national infrastructure that provides bioinformatics support to life science researchers. Our group already collaborates with BILS in ongoing metabolomics projects.

Implications

A recent systematic review concluded that *"Despite advances in treatment that have helped to improve outcomes for patients with RA, treatment goals, aspirations, and expectations are seldom met for both patients and physicians. RA continues to present a considerable human and economic burden. Novel treatment approaches for RA need to be tested for their ability to ameliorate contemporary unmet need."*[5] In Sweden, 45% of RA patients still have moderate/high disease activity, reflecting serious shortcomings in treatment and care also in Sweden. Our project thus addresses an unmet need in Sweden as

well as internationally. It constitutes a novel treatment approach that may have high cost benefit due to its low adverse event rate and low cost, in comparison with new and potent medications.

The nutritional status of patients with RA is poor and many ask for diet advice[19]. However, no evidence based dietary guidelines exist because of the paucity of well-conducted sufficiently large diet intervention trials. ADIRA will here contribute high-quality scientific evidence, being based on state-of-the art methodology in terms of diet composition, intervention delivery and evaluation of compliance.

References

[1]Scott DL et al. The course of established rheumatoid arthritis. Best Prect Res Clin Rheumatol 2007;21:943-67.

[2]Miossec P et al. Biomarkers and personalized medicine in rheumatoid arthritis: a proposal for interactions between academia, industry and regulatory bodies. Ann Rheum Dis 2011;70:1713-8.

[3]Barber CE, Smith A, et al. Best practices for cardiovascular disease prevention in rheumatoid arthritis: a systematic review of guideline recommendations and quality indicators. Arthritis Care Res (Hoboken). 2015;67(2):169-79.

[4]Lindhardsen J, Ahlehoff O, et al. The risk of myocardial infarction in rheumatoid arthritis and diabetes mellitus: a Danish nationwide cohort study. Ann Rheum Dis. 2011;70(6):929-34.

[5]Choy E, Ganeshalingam K, et al. Cardiovascular risk in rheumatoid arthritis: recent advances in the understanding of the pivotal role of inflammation, risk predictors and the impact of treatment. Rheumatology (Oxford). 2014;53(12):2143-54.

[6]Elkan AC, Engvall IL, et al. Rheumatoid cachexia, central obesity and malnutrition in patients with low-active rheumatoid arthritis: feasibility of anthropometry, Mini Nutritional Assessment and body composition techniques. Eur J Nutr. 2009;48(5):315-22.

[7]Scott DL et al. Rheumatoid arthritis. Lancet 2010,376:1094-108.

[8]Nam JL et al. Current evidence for the management of rheumatoid arthritis with biological disease-modifying antirheumatic drugs. Ann Rheum Dis. 2010;69:976-86.

[9]Taylor PC et al. A structured literature review of the burden of illness and unmet needs in patients with rheumatoid arthritis: a current perspective. Rheumatol Int 2016, Jan 8.

[10]Forouzanfar MH et al. Global, regional, and national comparative risk assessment of 79 behavioural, enviromental and occupational, and metabolic risks or clusters of risks in 188 countries, 1990-2013: a systematic analysis for the Global Burden of Disease Study 2013. Lancet 2015;S0140-6736.

[11]Kalkan A et al. Costs of rheumatoid arthritis during the period 1990-2010: a register-based cost-of-illness study in Sweden. Rheumatology 2014;53(1)

[12]World Health Organization. Global Health Risks: mortality and burden of disease attributable to selected major risks. 2009 WHO, Geneva.

[13]O'Connor Á. An overview of the role of diet in the treatment of rheumatoid arthritis. Nutrition Bulletin 2014;39(1):74-88.

[14]Aho K, Heliovaara M. Risk factors for rheumatoid arthritis. Ann Med. 2004;36(4):242-51.

[15]Di Giuseppe D, Discacciati A, et al. Cigarette smoking and risk of rheumatoid arthritis: a dose-response meta-analysis. Arthritis Res Ther. 2014;16(2):R61.

[16]Di Giuseppe D, Bottai M, et al. Physical activity and risk of rheumatoid arthritis in women: a population-based prospective study. Arthritis Res Ther. 2015;17:40.

[17]Albers R et al. Monitoring immune modulation by nutrition in the general population: identifying and substantiating effects on human health. Br J Nutr 2013;110:S3-S30.

[18]Calder PC and P Yaqoob eds. *Diet, immunity and inflammation*. First ed. 2013, Woodhead Publ Ltd: Cambrigde UK.

[19]Calder PC et al. Inflammatory disease processes and interactions with nutrition*.* Br J Nutr 2009. 101(Suppl 1): p S1-45.

[20]Vaghef-Mehrabany E et al. Probiotic supplementation improves inflammatory status in patients with rheumatoid arthritis*.* Nutrition 2014;30(4):430-5.

[21]National Food Agency. Find your way to eat greener, not too much and be active. Kalmar 2015 ISBN: 978 91 7714 242 3

[22]Salminen E, Heikkilä S, et al. Female patients tend to alter their diet following the diagnosis of rheumatoid arthritis and breast cancer. Prev Med. 2002;34(5):529-35.

[23]Grimstvedt ME, Woolf K, et al. Lower Healthy Eating Index-2005 dietary quality scores in older women with rheumatoid arthritis v. healthy controls. Public Health Nutr. 2010;13(8):1170-7.

[24]Di Giuseppe D, Wallin A, et al. Long-term intake of dietary long-chain n-3 polyunsaturated fatty acids and risk of rheumatoid arthritis: a prospective cohort study of women. Ann Rheum Dis. 2014;73(11):1949-53.

[25]Hayashi H, Satoi K, et al. Nutritional status in relation to adipokines and oxidative stress is associated with disease activity in patients with rheumatoid arthritis. Nutrition. 2012;28(11–12):1109-14.

[26]Hagen KB et al. Dietary interventions for rheumatoid arthritis. Cochrane Database Syst Rev 2009(1): p. CD006400.

[27]Skoldstam L et al. An experimental study of a Mediterranean diet intervention for patients with rheumatoid arthritis. Ann Rheum Dis 2003. 62(3): p. 208-14.

[28]McDougall J, Bruce B, et al. Effects of a very low-fat, vegan diet in subjects with rheumatoid arthritis. J Altern Complement Med. 2002;8(1):71-5

[29]Sarzi-Puttini P, Comi D, et al. Diet therapy for rheumatoid arthritis: A controlled double-blind study of two different dietary regimens. Scand J Rheumatol. 2000;29(5):302-7.

[30]Miles EA, Calder PC. Influence of marine n-3 polyunsaturated fatty acids on immune function and a systematic review of their effects on clinical outcomes in rheumatoid arthritis. Br J Nutr. 2012;107 Suppl 2:S171-84.

[31]Alipour B, Homayouni-Rad A, et al. Effects of Lactobacillus casei supplementation on disease activity and inflammatory cytokines in rheumatoid arthritis patients: a randomized double-blind clinical trial. Int J Rheum Dis. 2014;17(5):519-27.

[32]Rontoyanni VG, Sfikakis PP, et al. Marine n-3 fatty acids for cardiovascular risk reduction and disease control in rheumatoid arthritis: "Kill two birds with one stone"? Curr Pharm Des. 2012;18(11):1531-42.

[33]Young SP et al. The impact of inflammation on metabolic profiles in patients with arthritis. Arthritis & Rheum 2013;65:2015-23.

[34]Lauridsen MB et al. ^{1}H NMR spectroscopy-based interventional metabolic phenotyping: a cohort study of rheumatoid arthritis patients. Proteome Res 2010;9:4545-53.

[35]Wang Z et al. ^{1}H NMR –based metabolomics analysis for identifying serum biomarkers to evaluate methotrexate treatment in patients with early rheumatoid arthritis. Exp Ther Med 2012;4:165-71.

[36]van Wietmarschen HA, Weidong Dai., Anita J. van der Kooij, et al. Characterization of Rheumatoid Arthritis Subtypes Using Symptom Profiles, Clinical Chemistry and Metabolomics Measurements. PLOS ONE 2012;7(9):e44331.

[37]Turesson C et al. Guidelines for the pharmaceutical management of rheumatoid arhritis. The Swedish Society of Rheumatology 2012; www.svenskreumatologi.se/riktlinjer.

[38]Tovar J et al. A diet based on multiple functional concepts improves cardiometabolic risk parameters in healthy subjects. Nutr & Metab 2012;9:29-40.

[39]Group EQ: EuroQol: a new facility for the measurement of health-related quality of life. Health Pol. 1990;16:199-208.

[40]Braizer J et al. The estimation of a preference-based measure of health from the SF-36. J Health Econ. 2002;21:271-92.

[41]Kharroubi S et al. Modelling covariates for the SF-6D standard gamble health state preference data using nonparametric Bayesian method. Soc Sci Med. 2007;64:1242-52.

Bilaga 2

Bilaga 2

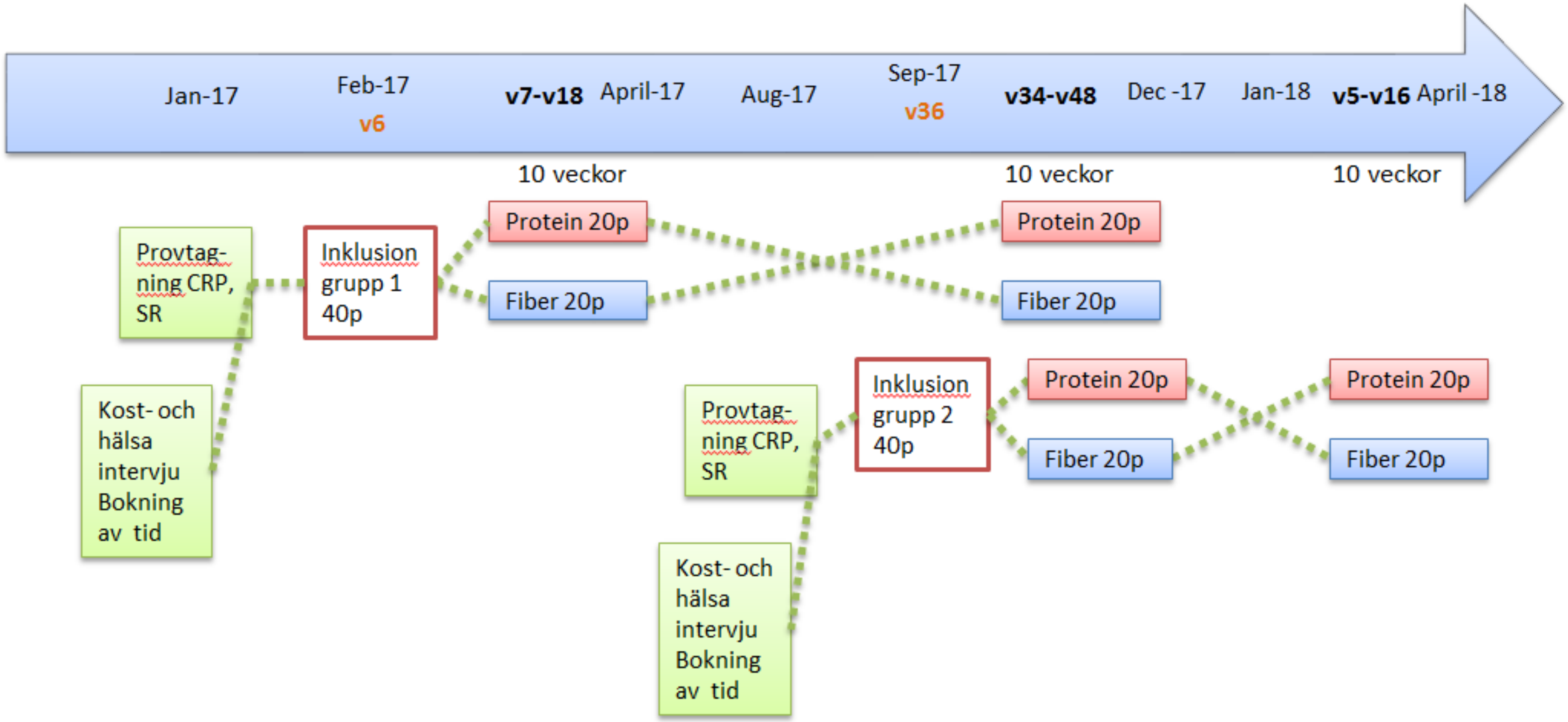

Figure 1. Flow chart of ADIRA

**Anti-inflammatory diet**	**Control diet, Western type**
Distributed weekly	*Distributed weekly*
3 fish meals (2 oily and 1 lean fish meals)<br>2 vegetarian meals, rich in prebiotics	5 meat or chicken meals
5 shots of probiotics	5 small servings of juice
5 servings of breakfast, rich in prebiotics (whole grain and fiber)	5 servings of breakfast (regular cereal and bread)
Fruit, berries and vegetables, rich in prebiotics and antioxidants	Sandwich and yoghurt snacks
Oliv-/ rapeseed oil for cooking	Butter for cooking
Margarine as spread	Butter for spread
Recipes and instructions	Recipes and instructions

Figure 2. Food bags for anti-inflammatory diet and control diet.
