# Supplementary material for: Effects on health-related quality of life in the randomized, controlled crossover trial ADIRA (Anti-inflammatory Diet In Rheumatoid Arthritis)
Source: PLoS One. 2021 Oct 14;16(10):e0258716. doi: 10.1371/journal.pone.0258716 (PMC8516209; doi:10.1371/journal.pone.0258716)
Supplement: Attachment — Submitted filename: Response to reviewers.docx [file pone.0258716.s007.docx]

REBUTTAL LETTER

MS ID#:  PONE-D-20-30996
Title:  Effects on health-related quality of life in the randomized, controlled crossover trial ADIRA (Anti-inflammatory Diet In Rheumatoid Arthritis)

Please find below our response to the comments from reviewers:

*Comments from Reviewers:*

Reviewer #1: I thank the authors for their response to reviewers' comments which has vastly improved the manuscript.

There are a few remaining issues which the authors have not revised but have instead offered explanations or refutations to justify their original position. I would like to comment on these with a view to coming to an agreement that is consistent with the standard practice for analysing and reporting randomised trials.

In the results table for a continuous outcome in a randomised trial, it is standard practice to report the means and standard errors of the continuous outcome in each group - not confidence intervals - followed by the between-group differences, confidence intervals for the between-group difference, and p-values for the hypothesis tests comparing outcomes in the groups. Please amend Table 3 and Table 4 to be consistent with this standard practice.

It is also not standard practice to perform hypothesis tests/report p-values for differences in baseline characteristics between arms in a randomised trial, as you have done in Table 2. Between-group differences at baseline are not expected to occur in this study design due to randomisation, and any occurrence thereof is purely by chance and therefore there's nothing you could meaningfully make of it. It would therefore be best to remove the p-value column and note (c) from Table 2. For the same reason, it is not standard practice in randomised trials to explore confounding by baseline covariates, because randomisation 'designs away' any known/unknown/potential confounding. Arguments have been made for exploring confounding in 'small' trials to deal with chance imbalance which is more likely when you have fewer observations; however, if a study is prospectively designed to answer a research question - including a sample size calculation which justifies the number of participants required - then it cannot be deemed 'small' and susceptible to chance imbalances to justify exploring for confounding.

I had previously commented that phrases such as "a trend towards significance" or "close to significant" should be avoided, and the authors responded by saying that such small p-values, in light of the small sample size, should not lead to a conclusion of no change. The authors chose to keep these statements and to indicate that something may be going on that could be evaluated with a larger sample size/stronger study design in the future. While I agree that a conclusion of no change would not be appropriate, the correct inference to be drawn from those p-values (given the parameters of the design of the study) is "no evidence of a difference". This is not the same as concluding no change or saying that there is no difference; rather, this is simply an interpretation of the statistical evidence for/against the hypothesis being tested. After presenting the whole discussion including issues around the small size of the study, a conclusion can be drawn that bears this evidence in mind when determining what it means or does not mean. Phrases such as "a trend towards significance" or "close to significant" when interpreting hypothesis tests are poor (and non-standard) practice that should be avoided entirely.

**Response: Thank you so much for your engagement and your valuable inputs. We have made all the changes suggested. However, to be able to discuss and evaluate also within-group differences, we chose to keep the confidence intervals for these estimates, but as you suggested, in addition we added the standard errors of the estimates.**
